# Vigorous Growing of Donor Plantlets by Liquid Overlay in Subcultures Is the Key to Cryopreservation of Endangered Species *Pogostemon yatabeanus*

**DOI:** 10.3390/plants11223127

**Published:** 2022-11-16

**Authors:** Hyoeun Lee, Haenghoon Kim

**Affiliations:** Department of Agricultural Life Science, Sunchon National University, Suncheon 57922, Republic of Korea

**Keywords:** activated charcoal, alternative vitrification solutions, gelling agent, regrowth medium, subculture medium

## Abstract

Cryopreservation is a unique option for the long-term conservation of threatened plant species with non-orthodox or limitedly available seeds. However, the wide application of cryopreservation for the protection of wild flora is hampered by some reasons: limits of source material available, difficulties in in vitro propagation, needs to re-optimize protocol steps for new species, etc. In this study, using an endemic and endangered Korean species, *Pogostemon yatabeanus*, we investigated subculture medium and supplements on in vitro growth of donor plants: medium strength, gelling agents, liquid overlay, plant hormones, and activated charcoal. Subculture conditions of each cycle tested significantly impacted on height and dry weight of subcultured donor plantlets. Among the treatments tested, the overlay of the liquid medium on top of gellan gum-gelled medium significantly increased the growth of shoots and roots. In the droplet-vitrification procedure, the survival and regeneration of cryopreserved shoot tips were critically impacted by the dry weight of donor plantlets (CORELL = 0.85~0.95) which was affected by the following subculture conditions. Moreover, every subsequent subculture cycle before cryopreservation positively or negatively impacted post-cryopreservation regeneration. This study highlights the vigor of donor plantlets for post-cryopreservation regeneration and provides practices for the revitalization of donor plants during subcultures.

## 1. Introduction

*Pogostemon yatabeanus* (Makino) Press [1] is an endemic and endangered species in Korea, which limitedly occurs in wetlands [2]. Cryopreservation, storing biological material in liquid nitrogen (LN, −196 °C), enables long-term preservation of genetic resources, keenly endemic and endangered species. Combined with in vitro technologies, cryopreservation offers a unique option for the long-term conservation and restoration of exceptional species (of non-orthodox or limitedly available seeds) [3]. When seed samples are limited in situ, in vitro culture and propagation of endemic and endangered species are helpful in preparing the explants for cryopreservation, restoration, and other uses [4]. In vitro culture of plant species is affected by diverse factors, including growth medium, gelling agents, growth regulators, culture conditions, etc. In vitro plantlets may not grow well spontaneously due to various reasons: an improper culture system, delaying of subculture interval, unskillful manipulations, or even with unknown causes. We developed an in vitro culture system of this species via in vitro seed germination, after dormancy-breaking, in vitro culture of nodal segments and cryopreservation of shoot tips of in vitro propagated plantlets [5,6].

The triangle of cryopreservation would be plant material, protocol, and manipulation (operational skill). In terms of plant material, numerous studies investigated genotype-dependent various responses [7], and a few kinds of literature examined the effect of donor plants and the selection of appropriate explants [4,7,8]. However, the importance of healthy donor plants and the choice of proper explants for cryopreservation have been pointed out [9,10]. To our knowledge, there is no suitable reference to the subculture medium. 

The protocol is the second but the central area of cryopreservation studies. Though there is no relevant data, manipulating protocol and person-to-person dependence is a big challenge. Thus more easy-handling protocols, such as the V- or D-cryoplate method, have widespread rapidly [11]. Indeed, skilled manipulation not only mattered in the cryopreservation procedure but also in preparing plant materials. As a solution-based vitrification method, droplet-vitrification (DV) is a multi-stage procedure with several factors from stage (1) material preparation to (2) pre-LN (preculture, osmoprotection, cryoprotection with vitrification solution), (3) LN (cooling, rewarming, unloading), and (4) post-LN (regrowth). However, most literature focuses on optimizing pre-LN stages, such as sucrose preculture concentration and PVS2 duration. In addition to the manipulation in pre-LN, LN, and post-LN stages, manipulation of mother plantlets and preparation of explants also need comprehensive skills. To prepare the explants for cryopreservation, investigators must propagate the in vitro plantlets under an already established procedure, depending on the species and type of organs. However, the growth of donor plants may be affected by diverse options of in vitro culture conditions, such as media, growth regulators, gelling agents, environmental conditions, and manipulation skills. 

The previous study on *P. yatabeanus* shoot tips optimized the latter three stages (stages 2–4) using a systematic approach. LN regeneration increased from below 20% to 73% and reached the highest of 90% with a stepwise regrowth medium [5,6]. Though DV methods have been implemented in crop collections, their wide application to threatened plant species is hampered by several reasons: a limited amount of starting material, non-optimized protocols for their in vitro propagation, the absence of the uniform cryopreservation protocol applicable to diverse gene pools, due to the extensive spectrum of physiological responses to cryopreservation stress observed among species [3]. The conditions of each stage should be optimized de novo for every target species [12], which requires many explants. Yet, in vitro propagation remains challenging in such species of limited source material for the experiments [13]. 

In this study, we investigated the effect of material preparation (stage 1) via diverse options of subculture medium and supplements to integrate the cryopreservation protocol using *P. yatabeanus* shoot tips. This study highlights the importance of subculture media and supplements that produce vigorously growing donor plants as source material for cryopreservation.

## 2. Results

### 2.1. Subculture Medium and Supplements in the Last Subculture Cycle (S-0)

#### 2.1.1. Effect of Subculture Medium and Supplements on In Vitro Growth of Donor Plants 

**Figure 1:**
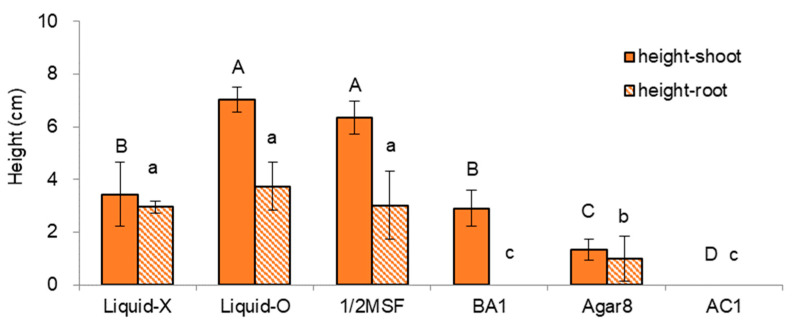
Plant height (cm) of in vitro grown *P. yatabeanus* plantlets with six treatments of subculture medium and supplements for five weeks. Liquid-X: MSF, gellan gum 3.0 g L^−1^, Liquid-X; Liquid-O: MSF, gellan gum 3.0 g L^−1^, Liquid-O; 1/2MSF: half-strength MSF, gellan gum 3.0 g L^−1^, Liquid-X; BA1: MSF, gellan gum 3.0 g L^−1^, Liquid-X, benzyl adenine (BA) 1 mg L^−1^; Agar8: MSF, agar 8.0 g L^−1^, Liquid-X; AC1: MSF, gellan gum 3.0 g L^−1^, Liquid-X, activated charcoal (AC) 1 g L^−1^. Means with the same letters (A–D and a–c) in each graph are not significantly different by Least Significant Difference Test (*p* < 0.05).

**Figure 2:**
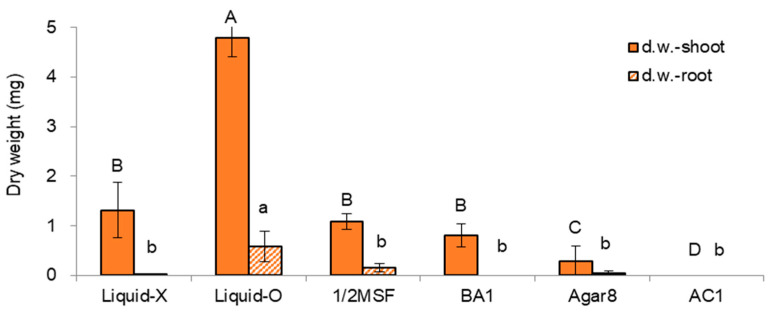
Dry weight (mg) of in vitro grown *P. yatabeanus* plantlets with six treatments of subculture medium and supplements for five weeks. Liquid-X: MSF, gellan gum 3.0 g L^−1^, Liquid-X; Liquid-O: MSF, gellan gum 3.0 g L^−1^, Liquid-O; 1/2MSF: half-strength MSF, gellan gum 3.0 g L^−1^, Liquid-X; BA1: MSF, gellan gum 3.0 g L^−1^, Liquid-X, benzyl adenine (BA) 1 mg L^−1^; Agar8: MSF, agar 8.0 g L^−1^, Liquid-X; AC1: MSF, gellan gum 3.0 g L^−1^, Liquid-X, activated charcoal (AC) 1 g L^−1^. Means with the same letters (A–D and a,b) in each graph are not significantly different by Least Significant Difference Test (*p* < 0.05).

The nodal sections were subcultured in the last cycle before cryopreservation in six variants of subculture medium and supplements (standard + 5 alternative conditions) for five weeks. Among the treatments tested, the overlay of liquid hormone-free MS medium (MSF, [14]) on top of gellan gum-gelled MSF medium (Liquid-O) produced significantly higher and heavier shoots. However, no significant differences in height and dry weight of the root part among the treatments tested (Figure 1 and Figure 2).

Liquid-O produced significantly higher shoot length and dry weight (7.0 cm and 4.8 mg) over the no liquid overlay condition (Liquid-X, 3.4 cm, and 1.3 mg), which is two-fold higher and three-fold heavier than the Liquid-X. Liquid-O also significantly increased the root dry weight (0.01 vs. 0.58 mg/plantlet). Indeed, We noticed no signs of hyperhydricity (physiological malformation, low lignification, or impaired stomatal function) in Liquid-O. Even the ratio of dry weight to fresh weight (DW/FW) in Liquid-O treatment was slightly higher than that of Liquid-X: 0.11 vs. 0.10. In addition, the ratio of shoot dry weight to fresh weight (DW/FW) correlated with shoot height (CORREL = 0.88) and dry weight (CORREL = 0.66) among the treatments, which indicates the more vigorous growth, the higher DW/FW ratio.

Though 1/2MSF resulted in a two-fold higher shoot length (6.4 cm vs. 3.4 cm), dry weight was even lower than the standard condition of MSF (Liquid-X) (1.1 mg vs. 1.3 mg). Application of benzyl adenine (BA) 1 mg L^−1^ (BA1) produced numerous shoots (10–12), but the length and dry weight of individual shoots were slightly decreased (2.9 cm and 0.8 mg). As a gelling agent, agar 8 g L^−1^ (Agar8) produced significantly more petite and slim shoots (1.3 cm and 0.3 mg) compared to the standard condition of gellan gum 3.0 g L^−1^ (Liquid-X, 3.4 cm, and 1.3 mg). Moreover, the addition of activated charcoal 1 g L^−1^ (AC1) stopped the growth of nodal sections. 

#### 2.1.2. Droplet-Vitrification Procedure

**Figure 3:**
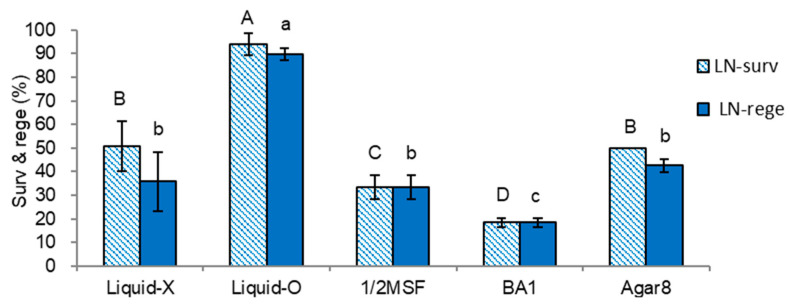
Effect of subculture medium and supplements on LN-survival (surv) and regeneration (rege) of *P. yatabeanus* shoot tips in regrowth steps of RM1(ammonium-free)-RM2-MSF. Liquid-X: MSF, gellan gum 3.0 g L^−1^, Liquid-X; Liquid-O: MSF, gellan gum 3.0 g L^−1^, Liquid-O; 1/2MSF: half-strength MSF, gellan gum 3.0 g L^−1^, Liquid-X; BA1: MSF, gellan gum 3.0 g L^−1^, Liquid-X, benzyl adenine (BA) 1 mg L^−1^; Agar8: MSF, agar 8.0 g L^−1^, Liquid-X. Means with the same letters (A–D and a–c) in each graph are not significantly different by Least Significant Difference Test (*p* < 0.05).

Node cutting-induced shoot tips from the last cycle of subculture (S-0) medium and supplements tested (Section 2.1.1.) were subjected to the standard droplet-vitrification procedure. The last subculture (S-0) medium and supplements significantly affected the survival and regeneration of cryopreserved shoot tips. Similar to the pattern of plant height and dry weight of the plantlets in Figure 1 and Figure 2, Liquid-O produced the highest LN survival and regeneration (93.9% and 89.7%) with a sequential regrowth medium of RM1(ammonium-free medium)-RM2-MSF (Figure 3). Shoot tips subcultured on gellan gum-gelled standard MSF medium without liquid overlay (Liquid-X) produced 50.8% survival and 35.8% regeneration after cryopreservation. However, shoot tips subcultured on 1/2MSF and BA1 had significantly lower LN survival and regeneration compared to the standard condition (Liquid-X). LN survival and regeneration of shoot tips subcultured on agar-gelled MSF medium (Agar8) was similar to the standard condition (Liquid-X). AC1 did not test since the samples were unavailable.

**Figure 4:**
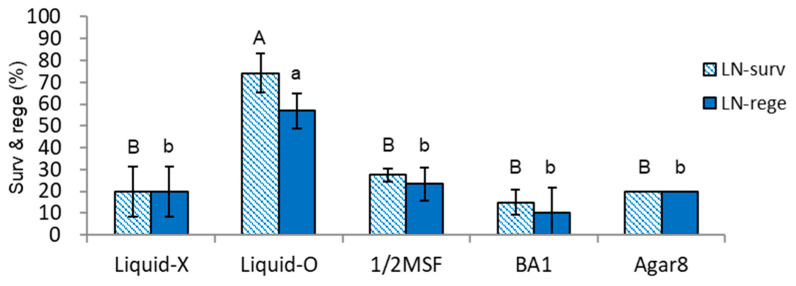
Effect of subculture medium and supplements on LN-survival (surv) and regeneration (rege) of *P. yatabeanus* shoot tips in regrowth steps of RM2 (ammonium-containing medium)-RM2-MSF. Liquid-X: MSF, gellan gum 3.0 g L^−1^, Liquid-X; Liquid-O: MSF, gellan gum 3.0 g L^−1^, Liquid-O; 1/2MSF: half-strength MSF, gellan gum 3.0 g L^−1^, Liquid-X; BA1: MSF, gellan gum 3.0 g L^−1^, Liquid-X, benzyl adenine (BA) 1 mg L^−1^; Agar8: MSF, agar 8.0 g L^−1^, Liquid-X. Means with the same letters (A,B and a,b) in each graph are not significantly different by Least Significant Difference Test (*p* < 0.05).

In a previous study [6], regrowth with standard MS medium (ammonium-containing) was harmful to the regeneration of LNC and LN shoot tips, possibly due to ammonium-induced oxidative stress. Therefore, we tried to investigate this regrowth option to compare the effect of the subculture medium. When the sequential regrowth medium of RM2 (initially ammonium-containing MS medium)-MS2-MSF was applied to the variants of subculture medium and supplements tested, LN survival and regeneration was significantly decreased, on average 18% lower, compared to those of RM1 (initial ammonium-free medium)-MS2-MSF (Figure 4). Especially in Liquid-O, LN survival and regeneration in RM2-RM2-MSF decreased by 19.6% and 32.9%, respectively, than those of RM1-RM2-MSF. All other treatments showed marginal LN regenerations, implying that the initial ammonium-containing regrowth medium is consistently harmful to any shoot tips of subculture conditions.

The regeneration of cryopreserved (LN) shoot tips was highly correlated with the dry weight of shoots and roots (Figure 2) among the variants of subculture medium and supplements tested, both in initial ammonium-free (RM1-RM2-MSF, Figure 3) (CORELL = 0.85–0.90) or ammonium-containing regrowth medium (RM2-RM2-MSF, Figure 4) (CORELL = 0.95–0.98, Table 1). The dry weight of subculturing donor plantlets is a more suitable indicator for cryo-tolerance than plant height. 

### 2.2. Revitalization of Donor Plants through the Subsequent Subcultures

#### 2.2.1. Selection of Three Subculture Variants

**Figure 5:**
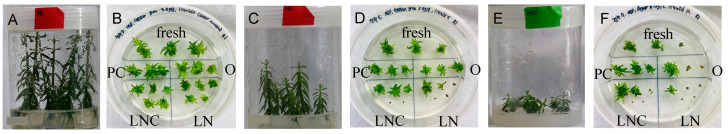
In vitro subcultured *P. yatabeana* plantlets for five weeks in the last subculture cycle before cryopreservation (C-0 cycle, (**A**,**C**,**E**)) and regrowth of subsequently cryopreserved shoot tips (**B**,**D**,**F**) after four weeks in regrowth medium of RM1 (ammonium-free MS)-RM2-MSF. Subculture condition for (**A**,**B**) was Gell+Liquid-O; C and D, Gell + Liquid-X; (**E**,**F**), Agar + Liquid-X. (for (**B**,**D**,**F**)) fresh, fresh control shoot tips; PC, preculture with 10% sucrose for 31 h; OP, PC and osmoprotected with C4–35% for 40 min; LNC, PC-OP and cryoprotected (CP) with A3–80% ice for 60 min, but not cryopreserved; LN, PC-OP-CP and cryopreserved (LN). OP, LNC, and LN shoot tips were unloaded with 30% sucrose solution for 40 min before regrowth.

Based on the observations in Figure 1, Figure 2, Figure 3 and Figure 4, after the repetitive standard subculture cycles (S-1), nodal sections were subjected to the last subculture before cryopreservation (S-0), with three variants of subculture medium for five weeks: Gell + Liquid-O (A), Gell + Liquid-X (C); Agar + Liquid-X (E in Figure 5). These three treatments represent vigorously, moderately, and slowly grown plantlets, respectively, among the treatments tested. 

Following the treatments of three subculture variants, nodal section-induced shoot tips were subjected to a standard droplet-vitrification procedure. Shoot tips after each step of precultured (PC), osmoprotected (OP), cryoprotected control (LNC), and cryopreserved (LN) were regrown in RM1 (ammonium-free MS)-RM2-MSF (Figure 5B,D,F). In vigorously grown plantlets (Gell + Liquid-O, Figure 5A), all the shoot tips from fresh control, PC, OP, LNC, and LN (Figure 5B) were regrown well. In moderately grown plantlets (Gell + Liquid-X, Figure 5C), though there was no difference in fresh control, PC, and OP shoot tips, shoot tips of LNC and LN were slowly grown, compared to the vigorously grown plantlets (Figure 5B), and about half shoot tips failed to regenerate. In slowly grown plantlets (Agar + Liquid-X, Figure 5E), shoot tips of OP, LNC, and LN were slowly grown, and only 1–2 shoot tips succeeded in regenerating. 

This result highlights that the growing pattern of donor plantlets in the last subculture cycle (S-0) critically affected subsequent LN regeneration. Additionally, this sensitivity-driven decline started at cryoprotection (LNC) or even osmoprotection (OP), eventually resulting in lower LN regeneration with the same standard droplet-vitrification procedure.

#### 2.2.2. In Vitro Growth of Donor Plantlets during the Subsequent Subculture Cycles 

In vitro plantlets subcultured with previously selected three subculture conditions in Figure 5 (Node + Gell + Liquid-O, Node + Gell + Liquid-X, Node + Agar + Liquid-X as S-1 cycle) were subsequently subjected to three subculture treatments to compare the efficiency of the revitalization of donor plants in the last subculture cycle before cryopreservation (totally nine conditions). The last three subculture (S-0 cycle) conditions consist of Apical + Gell + Liquid-O (apical section as an explant + gellan gum 3.0 g L^−1^ + overlay liquid MSF medium on top of gelled medium), Node + Gell + Liquid-O (nodal section + gellan gum 3.0 g L^−1^ + overlay liquid MSF medium), and Node + Gell + Liquid-X (nodal section + gellan gum 3.0 g L^−1^ + no liquid overlay). 

**Figure 6:**
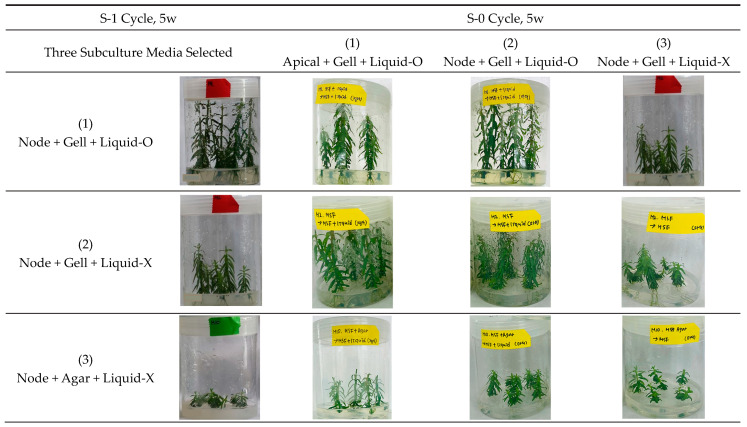
Re- and de-vitalization of donor plantlets of three subculture conditions from the S-1 cycle through the subsequent subculture of three subculture treatments in the S-0 cycle (total of nine states). The growth in the three subculture conditions of the S-1 cycle was significantly changed by the following three conditions of the S-0 cycle.

The growing pattern in the S-0 cycle (the last subculture cycle before cryopreservation) following the corresponding S-1 cycle (before the S-0 cycle) was influenced by the subculture combinations of both S-1 and S-0 cycle: dependent on both the vigor in previous S-1 cycle and subsequent subculture conditions in S-0 cycle (Figure 6). As noted in Figure 5, the vigor or growing pattern in the S-1 cycle was critically influenced by the combination of liquid overlay (Liquid-O vs. Liquid-X) and gelling agent (gellan gum vs. agar). Vigorisity of the plantlets subsequently subcultured in the S-1→S-0 cycle was dominantly affected by the order of Gell + Liquid-O > Gell + Liquid-X > Agar + Liquid-X. The order of Apical + Gell + Liquid-O influenced revitalization of these plantlets in the S-0 cycle > Node + Gell + Liquid-O > Node + Agar + Liquid-X, which indicates that the combination of inoculating apical section and liquid overlay on top of gellan gum-gelled medium is the choice for the facilitated revitalization of donor plants during subcultures.

Inoculation of apical sections associated with Gell + Liquid-O in the S-0 cycle (Apical + Gell + Liquid-O) effectively vitalized the slowly grown plantlets from the S-1 cycle (Node + Gell + Liquid-X, Node + Agar + Liquid-X). Whereas non-effective for the vigorously grown plantlets (Node + gell + Liquid-O), which implies that it reached the maximal performance in this subculture system. In addition, very slowly grown plantlets in the S-1 cycle (Node + Agar + Liquid-X) did not sufficiently revitalize even with Apical + Gell + Liquid-O in S-0, which means additional subculture cycles are needed to restore the plantlets before cryopreservation.

#### 2.2.3. Droplet-Vitrification Procedure Following the Subsequent Subculture Cycles

**Figure 7:**
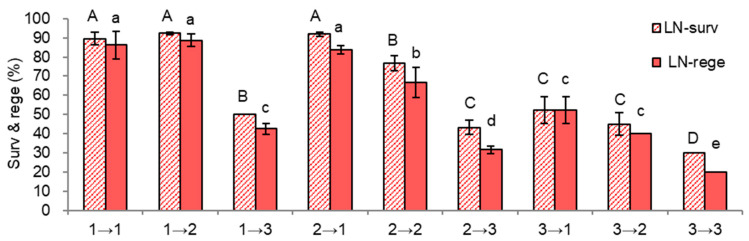
Effect of the combination of sequential subculture variants in S-1→ S-0 cycle on survival (surv) and regeneration (rege) of cryopreserved (LN) *P. yatabeanus* shoot tips. S-1 cycle; 1 (Node + Gell + Liquid-O), 2 (Node + Gell + Liquid-X), 3 (Node + Agar + Liquid-X)→S-0 cycle; 1(Apical + Gell + Liquid-O), 2(Node + Gell + Liquid-O), 3 (Node + Gell + Liquid-X). Means with the same letters (A–D and a–e) in each graph are not significantly different by Least Signif-icant Difference Test (*p* < 0.05).

After the subsequent subculture cycle of S-1→ and S-0 combinations (a total of nine variants in Figure 6), nodal section-induced shoot tips were subjected to cryopreservation using a standard droplet-vitrification procedure. The sequential subculture combinations of the S-1 and S-0 cycles impacted the survival and regeneration of cryopreserved shoot tips. Additionally, Gell + Liquid-O (1→1, 1→2, 2→1) both in S-1 and S-0 is critical for higher survival and regeneration of cryopreserved shoot tips (Figure 7). The higher LN survival (89.2–92.3%) and regeneration (83.8–88.7%) were produced only from those vigorously grown plantlets with a combination of Gell+Liquid-O in Figure 6.

LN regeneration of 2→1 (Node + Gell + Liquid-X in S-1→Apical+Gell+Liquid-O in S-0, 83.8%) was increased by 48%, compared to 2→3 (Node + Gell + Liquid-X in S-1→Node + Gell + Liquid-X in S-0, 35.8%) (Figure 7). Likewise, LN regeneration of 2→2 (Node + Gell + Liquid-X in S-1→Node + Gell + Liquid-O in S-0, 66.7%) was increased by 31%, compared to 2→3. Additionally, LN regeneration of 3→1 (Node+Agar+Liquid-X in S-1→Apical + Gell + Liquid-O in S-0, 55.2%) was increased by 13%, compared to the 3→2.

While, LN regeneration of 1→3 (Node + Gell + Liquid-O in S-1→Node + Gell + Liquid-X in S-0, 42.5%) was decreased by 47%, compared to 1→2 (Node + Gell + Liquid-O in S-1→Node + Gell + Liquid-O in S-0, 89.7%). Likewise, LN regeneration of 2→3 (Node + Gell + Liquid-X in S-1→Node + Gell + Liquid-X in S-0, 31.7%) was decreased by 35%, compared to 2→2 (Node + Gell + Liquid-X in S-1→Node + Gell + Liquid-O in S-0, 66.7%). Additionally, LN regeneration of 3→3 (20.0%) was decreased by 20%, compared to 3→2 (40%). This study implies that the conditions of every subculture cycle significantly affected the vigor of subcultured plantlets and eventually impacted post-cryopreservation regeneration; hence appropriate manipulation of donor plantlets is important for cryopreservation. 

Table 2 summarizes the mean survival and regeneration of LN shoot tips following the sequential subculture combinations of 3 (S-1) by 3 (S-0). Both treatments of Node + Gell + Liquid-O in S-1 and Apical + Gell + Liquid-O in the S-0 cycle produced the highest LN regeneration of 72.4% and 74.1%, respectively. The lowest LN regeneration rates of 37.4% and 31.4% were obtained from Node + Agar + Liquid-X in S-1 and Node + Agar + Liquid-X in the S-0 cycle. In comparison, 60.7% and 65.1% LN regeneration was determined by Node + Gell + Liquid-X in S-1 and Node + Gell + Liquid-O in the S-0 cycle, respectively. This result indicates that the conditions in the S-0 cycle (the last subculture) are slightly more influential on LN regeneration than those in the S-1 cycle (the previous subculture). Hence, sequential subcultures of donor plantlets with well-established appropriate conditions are prerequisites for higher LN regeneration of *P. yatabeanus* shoot tips.

## 3. Discussion

### 3.1. In Vitro Subculture and Propagation System

In vitro culture of plant material is a routine procedure for the initiation and preparation of explants in the cryopreservation of crops and wild species. In vitro approaches are essential, especially for endangered species with limited source materials [3]. Nodal sections are a valuable source for in vitro preparation of donor plants for cryopreservation since they are genetically stable and easy to manipulate. Like general plant tissue culture, subculturing of donor plantlets may be affected by diverse factors, such as source material, growth medium, culture conditions, etc. 

Gelling agents may affect the physiochemical characteristics of the culture medium by differences in elemental and organic impurities and diffusion rate of nutrients [15,16]. Different gelling agents are available commercially, including agar, gellan gum, gelrite, etc. Gellan gum is a polysaccharide produced from bacterial species [17]. As a gelling agent, gelrite has lower mineral content, fewer impurities of organic substances [18,19], better water availability [20], facilitated diffusion of inhibitive molecules such as phenols [21], compared to agar and thus gets advantages to stimulate the growth and development of in vitro plants: micropropagation and microtuberization [22], shoot multiplication [23], germination [24], conversion of polyembryoids into plantlets [25], and callus induction and roots development [16]. The immobilization of up to 30% MS medium salts may be an reason for agar’s inferiority over gerlite [26]. However, agar [27] or a mixture of gelrite and agar [28] produced better results over gelrite, which indicates the responses are species- and genotype-specific. Gelrite notably resulted in hyper-hydricity, primarily BA was associated with [29]. In this study, gellan gum was superior to agar on in vitro growth but did not significantly affect LN regeneration.

Liquid overlay on gelled medium displays solid and liquid medium advantages and eliminates the disadvantages of both systems [30]. Liquid overlay on gelled medium improved embryogenic initiation in *Pinus taeda* by allowing nutrient replenishment and adjustment of pH and hormones [31] and stimulating the multiplication of ginger [32]. Liquid overlay on top of the gelled medium produced significantly higher length and heavier dry weight of potato shoots and roots, compared to the other options, i.e., gelled (solid), wall-supported liquid, and static liquid medium (unpublished data). Though the mechanism of liquid overlay is not precise, the liquid overlay may first replenish beneficial substances and dilute any inhibitory substances [30,31]. Secondly, as in this study, it may promote plant growth by directly contacting stems with a liquid medium (water, sucrose, minerals, and other substances). Additionally, thirdly, it may change/modulate the osmotic potential of gelled medium and explants/plantlets and increase the dry weight by facilitating metabolite assimilation, respiration, and possibly photosynthesis. The second hypothesis may be feasible, particularly when the root system and its function were not sufficiently developed. However, our preliminary experiments with *P. yatabeana* plantlets may support the third hypothesis since a liquid overlay using sterilized distilled water (not MS medium) on top of the gelled medium was effective over Liquid-X (data not provided). Hence, the contribution of sucrose uptake and gross production to dry weight increase needs to be investigated [33]. In addition to in-depth analysis, further wide application of liquid overlay with other species is necessary since the species lives around wetlands. 

Practically, liquid overlay should be applied after 10–14 days of inoculation to avoid the hyperhydration of explants in *P. yatabeana* plantlets. If it is applied at the time of inoculation, the node cuttings are slightly turned brittle with a glassy appearance. In *Pinus taeda*, megagametophyte extrusion and somatic embryogenesis initiation were significantly increased when the liquid overlay was applied 14 days after inoculation, compared to 7 days or no application [31]. Hence, further integrated studies on the mechanism of liquid overlay are needed.

Activated charcoal (AC) has been used in tissue culture to improve cell growth and development by absorbing inhibitory compounds in the culture medium [34]. However, AC harmed the growth of *P. yatabeana* plantlets, possibly due to the absorption of needed substances or unknown reasons. However, it has a beneficial effect at the stage of in vitro germination and establishment. Other subculture conditions, such as medium strength, growth hormones, number of explants/vessels, and light intensity, did minor or adverse effects on in vitro growth and, eventually, LN regeneration (data not shown). 

### 3.2. Subculture Conditions and Post-Cryopreservation Regeneration 

Recovery of cryoprotected control (LNC) and cryopreserved (LN) explants may be affected by diverse factors, i.e., material preparation, pre-LN (pretreatments), LN (cooling and rewarming), and post-LN (regrowth) procedure. Although donor plantlets’ health is one of the critical factors in post-LN regeneration [10], little literature is available on this point. Generally, preconditioning of donor plants, such as cold acclimation and preparation of explants (apical/axillary, position, size), affect cryopreservation. Both cold acclimations of the mother plant at 5 °C for three weeks and increasing the last subculture duration before sampling were beneficial for the cryopreservation of apple shoot tips [35]. In comparison, smaller kiwi shoot tips (0.5–1 mm) from 2-week-old young shoots produced higher LN regeneration over larger shoot tips (1–2 mm) from 6-week-old donor plants acclimated at 4 °C [36]. Guerra et al. [7] noticed that pineapple shoot tips from 30 days subcultures had good morphoanatomical appearances and thus showed a higher survival rate compared to 45 days or 60 days subcultures. Subculture conditions, i.e., aeration of culture vessels, sparse planting density, and higher light intensity, helped produce healthy donor plants and thus significantly affected on recovery of LNC and LN shoot-tips in potatoes [8]. Reed [10] also pointed out that cultural conditions are as necessary as the cryopreservation protocol.

Since droplet-vitrification (DV) is a multi-stage procedure from material preparation to pre-LN, LN, and post-LN, it is necessary to optimize the process. Following the previous studies on LN, pre-, and post-LN stages in *P. yatabeana* shoot tips, we focused on preparing donor plants via subculturing in this study. Subculture medium and supplements significantly affected the growth of donor plantlets, consequently impacting the regeneration of LN shoot tips. Therefore, the primary hurdle of cryo-injury is the compatibility of shoot tips to cytotoxicity induced by cryoprotection with highly concentrated vitrification solution (VS). Additionally, the key to successful cryopreservation of *P. yatabeanus* is the vigorous growth of donor plantlets to cope with VS treatment if the appropriate protocol is available. However, the reason for slow growth may be diverse: improper culture system, delaying of subculture interval, unskillful manipulations, or even with unknown causes. In these cases, inoculation of apical nodal segments and overlay of the liquid medium on gelled medium has been applied spontaneously to revitalize the donor plants in our group. This study demonstrated that subculture medium and supplements significantly affected on in vitro growth of donor plants and further critically determined the LN regeneration (18.3–89.7%). In addition, sequential subculture conditions of each cycle were necessary, where the combination of gellan gum and liquid overlay (Apical + Gell + Liquid-O) was the best.

In vitro growth (height and dry weight) of *D. yatabeana* plantlets was affected by diverse factors of subculture medium and conditions tested. The differences in the growing pattern induced by one subculture cycle before cryopreservation subsequently affected the LN regeneration: the more vigorous growth, the higher LN survival and regeneration. The LN regeneration was highly correlated (CORRELL = 0.85–0.98) with the dry weight of shoots and roots among the subculture medium and supplements tested, both in initial ammonium-free (RM1-RM2-MSF) or -containing regrowth medium (RM2-RM2-MSF, Table 1). Our previous study pointed out that a three-step regrowth medium of RM1 (ammonium-free)-RM2 (GA_3_ and BA)-MSF (hormone-free medium) was critical for the regeneration of LNC and LN shoot tips in *P. yatabeanus* [6]. This result implies that the preparation of explants from vigorously growing donor plants with higher dry weight is the dominant factor for successful cryopreservation.

## 4. Materials and Methods

### 4.1. Plant Material, In Vitro Establish and Preparation of Mother Plants

In vitro plants of *Pogostemon yatabeanus* were grown from seeds and germinated in vitro following hot water treatment and three months of cold stratification as described previously [5,6]. 

Developed plants were propagated using single nodal segments (1.0~1.5 cm) via repeated (sequential) subcultures with hormone-free MS medium (MSF, [7]) with 30 g L^−1^ sucrose, 3.0 g L^−1^ gellan gum (MB Cell, Seoul, Korea) in 300 ml SPL culture vessels (90 × 40 mm, SPL Life Sciences, Gyeonggi-do, Korea). The liquid MSF medium (15 mL/vessel) was added on top of gellan gum-gelled medium at day 10 (Table 3 Liquid-O) (hereafter standard subculture condition) to facilitate the vigorous growth. The medium pH was adjusted to 5.8 before autoclaving at 121 °C for 25 min. Six single nodal segments were placed per vessel and kept at 25 °C under a 16/8 h light/dark photoperiod and one lamp (40 µE m^−2^ s^−1^) for six weeks.

### 4.2. Effect of Subculture Medium and Conditions in the Last Subculture Cycle (S-0)

#### 4.2.1. In Vitro Growth of Donor Plants

After repeated (sequential) 6-week subcultures with standard subculture conditions [node section + MSF medium + gellan gum 3.0 g L^−1^ + MSF liquid overlay on top of the gelled medium at day 10 (Node + Gell + Liquid-O)], nodal sections were subcultured one more cycle, as a final subculture before cryopreservation (S-0), in six variants of subculture medium and supplements (Table 3). During the experiment of each factor, other conditions remained the same as in the standard condition (indicated as “standard” in Table 3). The height (cm) and dry weight (mg) of shoots and roots were measured after five weeks. Dry weight was measured after 7 h at 75 °C ovens.

#### 4.2.2. Droplet-Vitrification Procedure

After repeated standard subcultures (Node + Gell + Liquid-O) and subsequent last cycle (S-0) in six variants of subculture medium and supplements for five weeks (Table 3), shoot tips (1.5 mm) were excised from 4–5-day-old single nodal sections which were cultured on MSF medium with 30 g L^−1^ sucrose, 3.0 g L^−1^ gellan gum in SPL culture vessels.

The cryopreservation procedure was adopted by Lee et al. [6]. Briefly, shoot tips were precultured in 10% sucrose (S–10%) for 31 h, osmoprotected with C4–35% (17.5% glycerol + 17.5% sucrose, *w*/*v*) for 40 min, then cryoprotected with alternative plant vitrification solution A3–80% (33.3% glycerol + 13.3% dimethyl sulfoxide + 13.3% ethylene glycol + 20.1% sucrose, *w*/*v*) for 60 min on ice. Then, explants were placed in ice-cold A3–80% on aluminum foil strips (7 × 20 mm) plunged directly in LN for a minimum of 1 h. Foil strips with shoot tips were rewarmed in 20 ml pre-heated (40 °C) unloading solution of 35% sucrose (S–35%) and kept for 40 min. The shoot tips were transferred to regrowth medium 1 [RM1, NH_4_NO_3_–free MS medium + 1 mg L^−1^ GA_3_ + 1 mg L^−1^ BA, 30 g L^−1^ sucrose, 3.0 g L^−1^ gellan gum] and cultured in the dark. After five days, explants were transferred to RM2 medium (the same as RM1 except for NH_4_NO_3_) and cultured under 40 µE m^−2^ s^−1^ for 23 days. The developed shoots were further transferred to MSF medium for two weeks.

### 4.3. Revitalization of Donor Plants through the Subsequent Subcultures

#### 4.3.1. In Vitro Growth of Donor Plants during the Subsequent Subculture Cycles, S-1→S-0

After sequential standard subcultures (S-2) with Node + Gell + Liquid-O, nodal sections were subcultured with three representative subculture conditions as S-1 subculture cycle: (1) Node + Gell + Liquid-O (vigorously grown), (2) Node + Gell + Liquid-X (moderately grown), (3) Node + Agar + Liquid-X (slowly grown). To investigate the efficiency of the revitalization of donor plants, nodal sections from the S-1 subculture cycle were subjected to three subsequent subculture conditions (a total of nine variants): (1) Apical + Gell + Liquid-O, (2) Node + Gell + Liquid-O, (3) Node + Gell + Liquid-X for five weeks as S-0, the last subculture cycle before cryopreservation (Table 4).

#### 4.3.2. Droplet-Vitrification Procedure Following the Subsequent Cycles 

After the sequential subculture of S-1→S-0, a growing pattern of subcultured plantlets was observed, and node-cutting-derived shoot tips from the S-0 cycle were subjected to the droplet-vitrification procedure described previously.

### 4.4. Recovery Assessment and Statistical Analysis

Survival was evaluated two weeks following cryopreservation by counting the number of shoot tips showing regrowth of greenish tissues. Regeneration was determined after six weeks when the shoots had developed into normal plantlets (≥8 mm) with fully expanded leaves and roots. Ten to 13 shoot tips were used per experimental condition, and the experiments were replicated three times. 

Data from all experiments were analyzed by analysis of variance (ANOVA) and Duncan’s multiple range test (*p* < 0.05) using SAS on Demand for Academics software (SAS Institute Inc., Cary, NC, USA). Results are presented as percentages with their standard deviations.

## Figures and Tables

**Table 1:** The correlation coefficient between height and dry weight of donor plantlets subcultured once in the last cycle before cryopreservation (nine variants) and regeneration of subsequently cryopreserved shoot tips.

Treatment Sets	Regrowth Steps (5d-3w-2w)	Height		Dry Weight	
		Shoots	Roots	Shoots	Roots
Subculture medium and supplements	RM1-RM2-MSF *	0.49	0.62	0.85	0.90
	RM2-RM2-MSF	0.75	0.74	0.95	0.98

* RM1, ammonium (NH_4_NO_3_)-free MS medium + 1 mg L^−1^ GA_3_ + 1 mg L^−1^ BA for five days; RM2, ammonium containing MS medium + 1 mg L^−1^ GA_3_ + 1 mg L^−1^ BA for three weeks; MSF, ammonium-containing MS medium without growth regulators for two weeks; d, days; w, weeks.

**Table 2:** The effect of subsequent subculture cycle of S-1→ and S-0 combinations on survival and regeneration of cryopreserved (LN) *P. yatabeanus* shoot tips.

Code	Subsequent Subculture Cycle		Mean of Three Subculture Combinations each (S-1→S-0)	
	S-1, 5 Weeks	S-0, 5 Weeks	LN Survival (%)	LN Regeneration (%)
1→1, 2, 3	Node + Gell + Liquid-O	Apical + Gell + Liquid-O Node + Gell + Liquid-O Node + Agar + Liquid-X	77.3	72.4
2→1, 2, 3	Node + Gell + Liquid-X	Apical + Gell + Liquid-O Node + Gell + Liquid-O Node + Agar + Liquid-X	70.6	60.7
3→1, 2, 3	Node + Agar + Liquid-X	Apical + Gell + Liquid-O Node + Gell + Liquid-O Node + Agar + Liquid-X	42.4	37.4
1, 2, 3→1	Node + Gell + Liquid-O Node + Gell + Liquid-X Node + Agar + Liquid-X	Apical+Gell+Liquid-O	77.9	74.1
1, 2, 3→2	Node + Gell + Liquid-O Node + Gell + Liquid-X Node + Agar + Liquid-X	Node+Gell+Liquid-O	71.3	65.1
1, 2, 3→3	Node + Gell + Liquid-O Node + Gell + Liquid-X Node + Agar + Liquid-X	Node+Agar+Liquid-X	41.1	31.4

The LN survival and regeneration in Figure 7 were transformed by combining three combinations of the S-1→ and S-0 cycle.

**Table 3:** Set of treatments to test the impact of subculture medium and conditions in S-0 cycle for five weeks on the growth of donor plantlets and regrowth of cryopreserved *P. yatabeanus* shoot tips.

No.	Medium	Gelling Agent	Liquid Overlay	Hormone	AC	Code	Remarks
		(g L^−1^)		(mg L^−1^)	(g L^−1^)		
1	MS	Gellan gum 3	Liquid-O	-	-	Liquid-O	
2	MS	Gellan gum 3	Liquid-X	-	-	Liquid-X	standard
3	MS	Agar 8	Liquid-X	-	-	Agar8	
4	1/2 MS	Gellan gum 3	Liquid-X	-	-	1/2 MSF	
5	MS	Gellan gum 3	Liquid-X	BA 1	-	BA1	
6	MS	Gellan gum 3	Liquid-X	-	AC 1	AC1	

1/2 MS, half-strength MS medium; Liquid-O, overlay of liquid hormone-free MS medium on top of the gellan gum-gelled hormone-free MS medium; Liquid-X, no liquid overlay; -, omission; BA, benzyl adenine; AC, activated charcoal.

**Table 4:** Set of sequential subculture conditions of donor plants, i.e., S-2 (repeated subcultures with Node+Gell+Liquid-O), S-1 (three subculture variants), S-0 (the last three subculture conditions to revitalize the donor plants from subculture S-1) in *P. yatabeanus* plantlets.

Repeated Standard Subcultures for PropAgation (S-2 *, 6w)	Subculture Treatment (Gelling Agent and Liquid Overlay, S-1 ** Cycle, 5w)	Subculture Treatment (Section, Gelling Agent, and Liquid Overlay) for Rejuvenation (S-0 ***, 5w)	Code (S-1→S-0 Cycle)
Node + Gell + Liquid-O	(1) Node + Gell + Liquid-O	(1) Apical + Gell + Liquid-O (2) Node + Gell + Liquid-O (3) Node + Gell + Liquid-X	1→1 1→2, standard 1→3
	(2) Node + Gell + Liquid-X	(1) Apical + Gell + Liquid-O (2) Node + Gell + Liquid-O (3) Node + Gell + Liquid-X	2→1 2→2 2→3
	(3) Node + Agar + Liquid-X	(1) Apical + Gell + Liquid-O (2) Node + Gell + Liquid-O (3) Node + Gell + Liquid-X	3→1 3→2 3→3

* S-2, repeated standard subculture conditions for propagation while maintaining the vigor of donor plants: nodal section + MSF medium + overlay of liquid MSF medium (15 mL/vessel) on top of the 3.0 g L^−1^ gellan gum-gelled medium at day ten after planting onwards. ** S-1, nodal sections were subcultured for five weeks with three conditions: Node + Gell + Liquid-O, Node + Gell + Liquid-X, or Node + Agar + Liquid-X. *** S-0, the last 5-week revitalization subculture before cryopreservation experiments: Apical + Gell + Liquid-O, Node + Gell + Liquid-O, or Node + Agar + Liquid-X.

## Data Availability

Not applicable.
